# Development, Immunogenicity and Protective Efficacy of an Associated Inactivated Vaccine Against Highly Pathogenic Avian Influenza and Newcastle Disease in Chickens

**DOI:** 10.3390/vaccines14080669

**Published:** 2026-08-01

**Authors:** Yeldos Myrzakhmetov, Nurika Assanzhanova, Sholpan Ryskeldinova, Aigerim Mailybayeva, Aigerim Sagymbayeva, Yerken Kozhamkulov, Ekaterina Yamanova, Rassul Sidikhov, Nurlan S. Kozhabergenov, Bekbolat Usserbayev, Kuanysh Zhekebekov, Sergazy Nurabayev, Kuandyk Zhugunissov, Nurlan Akmyrzayev

**Affiliations:** Research Institute for Biological Safety Problems, National Holding “QazBioPharm”, Gvardeiskiy 080409, Kazakhstan; y.myrzakhmetov@biosafety.kz (Y.M.); sh.ryskeldinova@biosafety.kz (S.R.); a.mailybayeva@biosafety.kz (A.M.); a.sagymbayeva@biosafety.kz (A.S.); e.kozhamkulov@biosafety.kz (Y.K.); e.yamanova@biosafety.kz (E.Y.); sidikovrb@mail.ru (R.S.); n.kozhabergenov@biosafety.kz (N.S.K.); b.usserbayev@biosafety.kz (B.U.); k.zhekebekov@biosafety.kz (K.Z.); s.nurabayev@biosafety.kz (S.N.); k.zhugunisov@biosafety.kz (K.Z.)

**Keywords:** avian influenza, Newcastle disease, associated inactivated vaccine, immunogenicity, protective efficacy, adjuvant, genotype

## Abstract

**Background**: The simultaneous circulation of highly pathogenic avian influenza (HPAI) H5N8 (clade 2.3.4.4b) and virulent Newcastle disease virus (NDV) genotype VII creates a cumulative risk for the poultry industry, rendering monovalent vaccination insufficient. This study aimed to develop and evaluate a combined inactivated vaccine against AIV H5N8 and NDV genotype VII based on local endemic strains. **Methods**: The vaccine was formulated as a water-in-oil emulsion (30:70) using Montanide™ ISA 78 VG. Evaluation included physicochemical characterization, safety assessment in chickens, immunogenicity evaluation via the hemagglutination inhibition (HI) test, and protective efficacy following challenge with virulent isolates. **Results**: The formulation demonstrated high stability and complete safety without adverse reactions. By 28 days post-immunization, the vaccine induced a robust, balanced humoral response with antibody titers reaching 9.10 ± 0.23 log_2_ against NDV and 9.00 ± 0.26 log_2_ against AIV, indicating no antigenic interference. Upon challenge, the vaccinated group exhibited 100% clinical protection and survival, compared to 100% mortality in controls. Furthermore, vaccination significantly reduced viral shedding, limiting horizontal spread. **Conclusions**: The developed combined inactivated vaccine demonstrates high safety, stability, and protective efficacy. It represents a promising candidate for comprehensive specific prophylaxis in endemic regions, effectively limiting horizontal pathogen transmission and mitigating economic losses in poultry production.

## 1. Introduction

In recent years, the situation of highly pathogenic avian influenza (HPAI) viruses remains one of the most significant epizootic challenges in the global poultry industry. Since 2020, the H5N8 subtype virus (clade 2.3.4.4b), and subsequently H5N1, has assumed a panzootic character, spreading across all continents and causing massive outbreaks both in commercial poultry production and among wild bird populations [1]. These epizootics are accompanied by record mortality rates, mandatory culling of flocks, and direct economic losses estimated at billions of US dollars annually [2]. The migration of waterfowl and semi-aquatic birds, whose routes pass through transcontinental corridors, remains a key driver of the global spread of HPAI, creating a constant threat of virus introduction to new territories and potential risks to human health [3].

In Europe, during the 2024–2025 season, the number of laboratory-confirmed HPAI cases among wild birds exceeded the figures of the three previous years and was 4.8 times higher than in 2023, reflecting a steady trend towards the intensification of the epizootic process [4]. In Kazakhstan, the epizootic situation regarding HPAI is also highly critical: in 2020, major outbreaks of the H5N8 subtype were recorded in the Almaty, Akmola, and Kostanay regions, accompanied by massive bird mortality and restrictions on the movement of poultry products [5,6].

Alongside avian influenza, Newcastle disease (ND) remains a significant challenge. Over the past decade, Newcastle disease virus (NDV) strains of genotype VII, characterized by high virulence, broad neurotropism, and the ability to cause epizootics with mortality rates reaching 90–100% in unvaccinated flocks, have become dominant across Eurasia and the Commonwealth of Independent States (CIS) [7]. Epizootiological analysis of isolates collected in Kazakhstan up to 2023 has confirmed the active circulation of virulent genotype VII strains (including subgenotypes VII.1 and VII.2), indicating the insufficient efficacy of current vaccination regimens—a finding consistent with observations in other countries facing a similar genotypic landscape [8].

A growing body of epizootiological data indicates that monovalent vaccination targeting either Newcastle disease virus (NDV) or avian influenza virus (AIV) exclusively may be insufficient in endemic regions where the co-circulation of both pathogens is documented [9]. This issue is particularly critical for regions with intensive poultry production, where the simultaneous spread of highly virulent genotype VII NDV strains and H5 subtype AIV poses a cumulative risk to epizootic stability [10]. Particular attention must be paid to the backyard poultry sector, which often remains outside systematic veterinary surveillance, exhibits high seroprevalence for both pathogens, and serves as a potential reservoir and epidemiological bridge for the transmission of infection between wild birds and commercial poultry operations [11]. Consequently, the development and evaluation of an inactivated combined vaccine capable of inducing simultaneous protective immunity against H5 subtype AIV and genotype VII NDV represents a strategic imperative. It is hypothesized that the use of such oil-based formulations will ensure clinical protection and reduce viral shedding, thereby helping to break epizootic chains in settings with limited biosecurity resources [12,13].

The aim of the present study was the whole-genome characterization of the epizootic NDV strain, along with the development and comprehensive laboratory evaluation of a combined inactivated vaccine against avian influenza (subtype H5) and Newcastle disease (genotype VII), including the analysis of its physicochemical properties and the assessment of its safety, immunogenicity, and protective efficacy. The data obtained will provide a scientific rationale for the potential of this vaccine formulation as a tool for comprehensive prophylaxis in regions with the concurrent circulation of both pathogens.

## 2. Materials and Methods

### 2.1. Library Preparation and Whole-Genome Sequencing of Newcastle Disease Virus

The Newcastle disease virus strain “PMV-1/chicken/Almaty/710/04”, isolated by researchers at the Institute of Microbiology and Virology of the Committee of Science, Ministry of Education and Science of the Republic of Kazakhstan, and maintained in the microorganism collection of the Research Institute for Biological Safety Problems (RIBSP), was used in this study. Viral mass propagation was carried out in the allantoic cavity of 9–11-day-old specific pathogen-free (SPF) chicken embryos obtained from the “Podmoklovo” breeding farm (Moscow, Russia). The embryos were inoculated via the allantoic route and incubated at (37.0 ± 0.5) °C for 72 h at 60–70% humidity. Upon completion of incubation, the embryos were chilled at (4 ± 2) °C for 12 h, after which the allantoic fluid was aseptically harvested. The initial infectivity titer of the resulting viral suspension was 9.70 ± 0.14 log_10_ EID_50_/mL.

cDNA library preparation was performed using the Ion Plus Fragment Library Kit (Thermo Fisher Scientific, Waltham, MA, USA) according to the manufacturer’s instructions. Purification and size selection of 350–500 bp fragments were carried out by agarose gel electrophoresis followed by extraction using the innuPREP DOUBLEpure Kit (Analytik Jena, Jena, Germany). Library amplification and quantification were performed using the Ion Plus Fragment Library Kit and the Ion Universal Library Quantitation Kit. To minimize potential errors associated with manual handling, rigorous quality control of the prepared libraries, including fragment size distribution and precise concentration assessment, was performed according to the manufacturer’s recommendations prior to template preparation.

Template preparation and chip loading were performed on the Ion Chef automated station using Ion 530 chips (Thermo Fisher Scientific). Whole-genome sequencing (WGS) was conducted on the Ion GeneStudio S5 platform. Primary data processing and export of files in FASTQ and UBAM formats were carried out using Torrent Suite v5.12 software.

#### Genome Assembly

Genome assembly was performed using the UGENE software package version 52. The input data consisted of NGS reads in FASTQ format. A reference-based assembly approach was employed, using the Newcastle disease virus genome sequence as the reference (NCBI accession: GU585905.1). Default alignment and assembly parameters recommended by the software were used. The quality of the genome assembly was subsequently assessed by evaluating the read coverage depth and mapping quality within the UGENE (version 50, Unipro, Novosibirsk, Russia) software, ensuring the generation of a high-confidence consensus sequence with >99% genome breadth of coverage.

### 2.2. Phylogenetic Analysis of Newcastle Disease Virus

For phylogenetic analysis, the complete genome sequence of the investigated strain “PMV-1/chicken/Almaty/710/04” was aligned with nucleotide sequences of other *Orthoavulavirus* representatives retrieved from the NCBI database. Phylogenetic analysis was performed using the neighbor-joining (NJ) method [14]. An optimal phylogenetic tree was constructed with bootstrap support values (1000 replicates) indicated at the nodes [15]. The tree is presented at a scale of 0.02, where branch lengths correspond to evolutionary distances calculated by the Maximum Composite Likelihood method and expressed as the number of nucleotide substitutions per site [16]. For each internal clade, the proportion of sites with an unambiguous base in at least one sequence is shown. For each internal clade, the proportion of sites with an unambiguous base in at least one sequence is shown. Evolutionary analysis was conducted using MEGA software (version 11.0, Institute for Genomics and Evolutionary Studies, Temple University, Philadelphia, PA, USA) [17].

### 2.3. Molecular and Genetic Characterization of Avian Influenza Virus

The antigenic component of the vaccine was the highly pathogenic avian influenza virus strain of the H5N8 subtype (“A/Wild goose/Kostanay/KZ/83/2021”, clade 2.3.4.4b), whose molecular and biological properties have been described in detail previously [5]. Viral mass propagation was performed in the allantoic cavity of 9–11-day-old specific pathogen-free (SPF) embryos, followed by incubation at (37.0 ± 0.5) °C for 48 h. The initial infectivity titer of the resulting viral suspension was 9.78 ± 0.14 log_10_ EID_50_/mL.

### 2.4. Inactivation of Virus Suspensions and Preparation of Emulsified Vaccine

Inactivation of the viral suspensions and verification of its completeness were performed according to our previously validated protocol [18,19], which included treatment with a 0.05% formaldehyde solution and three blind passages in SPF embryos to confirm the absence of residual virulence.

**Figure 1:**
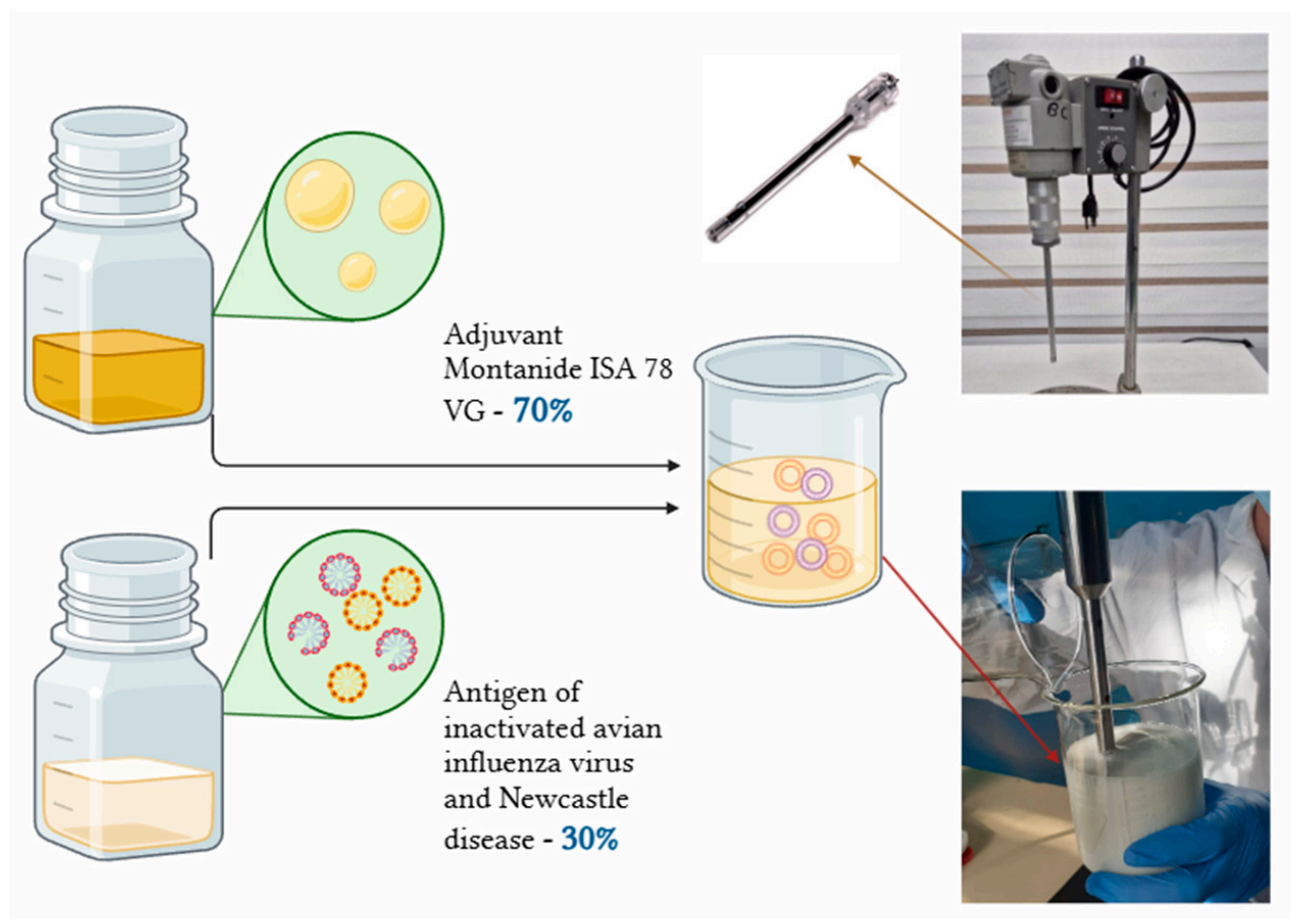
Schematic representation of the vaccine emulsification process. The diagram illustrates the gradual addition of the aqueous phase to the oil phase under continuous homogenization to form a stable water-in-oil (W/O) emulsion. (Image created using BioRender.com).

The adjuvanted vaccine was formulated by emulsification using Montanide™ ISA 78 VG (SEPPIC, Paris, France) as the adjuvant, whose efficacy and safety in poultry veterinary immunoprophylaxis have been previously confirmed [20]. The formulation was a water-in-oil (W/O) emulsion with a phase ratio of 30:70. Emulsification was performed by gradually adding the aqueous phase to the oil phase under continuous homogenization (2500 rpm for 30 min) until a stable homogeneous system was obtained (Figure 1). The finished product was tested for sterility and emulsion stability.

Quality control of the experimental batch of the combined inactivated vaccine against Newcastle disease and avian influenza was performed using a comprehensive set of physicochemical, biological, and technological parameters: sterility, safety, pH value, kinematic viscosity, emulsion system stability, and immunogenic activity.

The stability assessment of the experimental batch of the combined inactivated vaccine against Newcastle disease and avian influenza was conducted under long-term storage conditions at temperatures of (4 ± 2) °C and (25 ± 2) °C. Monitoring was based on physicochemical parameters: visual assessment for the absence of phase separation, measurement of kinematic viscosity, determination of the mean globule size and polydispersity index via dynamic light scattering, and a drop test to evaluate the emulsion’s resistance to spreading [21,22]. The evaluation criteria (absence of phase separation, kinematic viscosity, and globule size) were in accordance with WHO guidelines on vaccine stability evaluation [23] and established scientific standards for the quality control of water-in-oil emulsions in veterinary immunoprophylaxis [24].

### 2.5. Safety Evaluation of the Vaccine Preparation

The safety evaluation was performed on 28-day-old “Super Nick” egg-layer crossbred chicks obtained from SPF eggs. The seronegative status of the birds was confirmed by the hemagglutination inhibition (HI) test (titer < 1:4). The birds were kept in isolated vivarium boxes under controlled environmental conditions (temperature 22 ± 2 °C, relative humidity 45–55%) with unlimited access to feed and water. The safety assessment included daily clinical monitoring for 10 days following vaccine administration, with recording of general activity, appetite, feather condition, and the presence of local reactions. All animal procedures were performed in accordance with Directive 2010/63/EU [25]. The animal study was conducted under Protocol No. 1, dated 20 January 2021, approved by the Ethics Committee of the Research Institute for Biological Safety Problems.

### 2.6. Assessment of Vaccine Immunogenicity

Blood samples were collected from 28-day-old chicks prior to vaccination to obtain serum for determination of the baseline immune status. The vaccine was administered to the experimental group via the intramuscular route at a dose of 0.5 mL per bird. All serum samples, including those collected at 7, 14, 21, and 28 days post-immunization, were analyzed simultaneously by the hemagglutination inhibition (HI) test to minimize inter-plate variability. The immunogenicity study involved 40 chicks, which were randomly allocated into two groups of 20 birds each: vaccinated birds and unvaccinated birds (control). Specific antibody titers against NDV and HPAIV were determined by the HI test according to the methodological recommendations of the World Organisation for Animal Health (WOAH) [26]. The vaccine was considered immunogenic if at least 80% of birds in the experimental group demonstrated seroconversion with an antibody titer ≥1:16 (≥4 log_2_) to both viruses at 28 days post-vaccination.

### 2.7. Experimental Challenge and Study of Protective Efficacy

To evaluate the vaccine efficacy, chicks from the experimental (EG) and control (CG) groups were divided into two subgroups on day 28 post-immunization and challenged with virulent epizootic isolates: Newcastle disease virus (NDV) “PMV-1/chicken/Almaty/710/04” and avian influenza virus (AIV) subtype H5N8 “A/Wild goose/Kostanay/KZ/83/2021” (clade 2.3.4.4b).

Challenge was performed separately for each pathogen:

Subgroup EG-NDV (and the corresponding control subgroup C-NDV): challenged intramuscularly via the pectoral muscle with NDV at a dose of 10^5^ EID_50_ [27].

Subgroup EG-AIV (and the corresponding control subgroup C-AIV): inoculated intranasally with AIV at a dose of 10^6^ EID_50_ [28].

**Figure 2:**
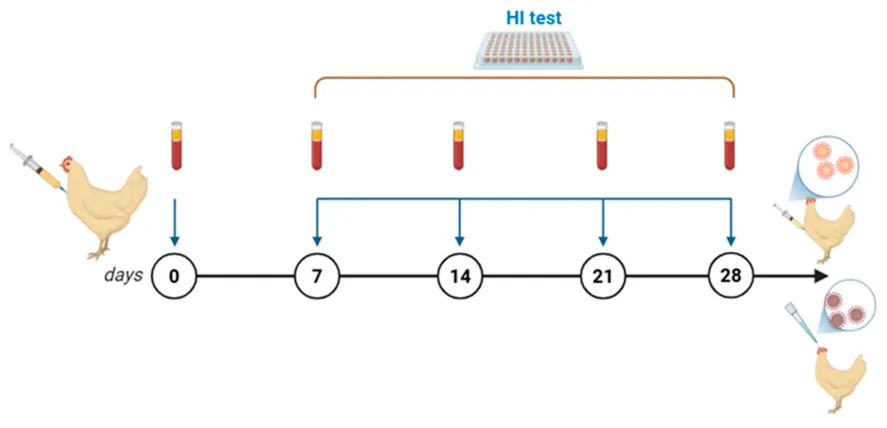
Chicken immunization and challenge schedule. The timeline depicts the primary vaccination of chicks at day 0, blood sampling for immune response evaluation at days 7, 14, 21, and 28, and the subsequent viral challenge at day 28, followed by a 10-day observation period. (Image created using BioRender.com accessed on 3 March 2026).

The total inoculum volume for each bird was 0.5 mL (Figure 2). The infectivity titers of the administered doses were verified by back-titration using specific pathogen-free (SPF) chicken embryos.

During the 10-day period following the challenge, daily clinical examinations of the birds were conducted using a standardized quantitative scale to assess the severity of the infectious process: 0 points—clinically healthy bird; 1 point—moderate anorexia or decreased water consumption; 2 points—mild respiratory distress or characteristic greenish diarrhea; 3 points—severe depression, neurological signs, or agonal state; 4 points—death. To minimize animal suffering, humane endpoints were strictly applied. Birds reaching a clinical score of 3 (severe depression, neurological signs, or agonal state) were immediately euthanized by intravenous administration of barbiturates in accordance with the American Veterinary Medical Association (AVMA) guidelines for the euthanasia of animals. Birds that reached the humane endpoint were considered as mortality events in the survival analysis.

For each experimental group, the mean clinical score (MCS) was calculated as an integral indicator of disease severity. Individuals that maintained a score of 0 throughout the observation period were classified as fully protected. The protection rate (%) was calculated using the formula: Protection (%) = (Number of surviving birds/Total number of challenged birds) × 100, according to the method described by [28]. In accordance with international standards for the evaluation of veterinary vaccine efficacy [29], the vaccine was deemed efficacious if it demonstrated a protective efficacy of at least 50% in the vaccinated group compared to the control group.

To evaluate virus shedding in vaccinated birds, tracheal and cloacal swabs were collected from five birds per group at 3, 5, and 7 days post-challenge. The samples were inoculated into embryonated chicken eggs for virus isolation, and viral infectious titers (lg EID_50_/mL) were determined.

### 2.8. Statistical Analysis

Antibody titers were log_2_-transformed and expressed as mean log_2_ titer ± SEM. Kinetics of the antibody responses were analyzed using two-way RM ANOVA followed by Sidak’s multiple comparisons test. Survival and seroconversion rates were compared using Fisher’s exact test. Statistical analyses were performed in GraphPad Prism 8.0, with statistical significance set at *p* < 0.05.

## 3. Results

### 3.1. Phylogenetic Analysis

**Figure 3:**
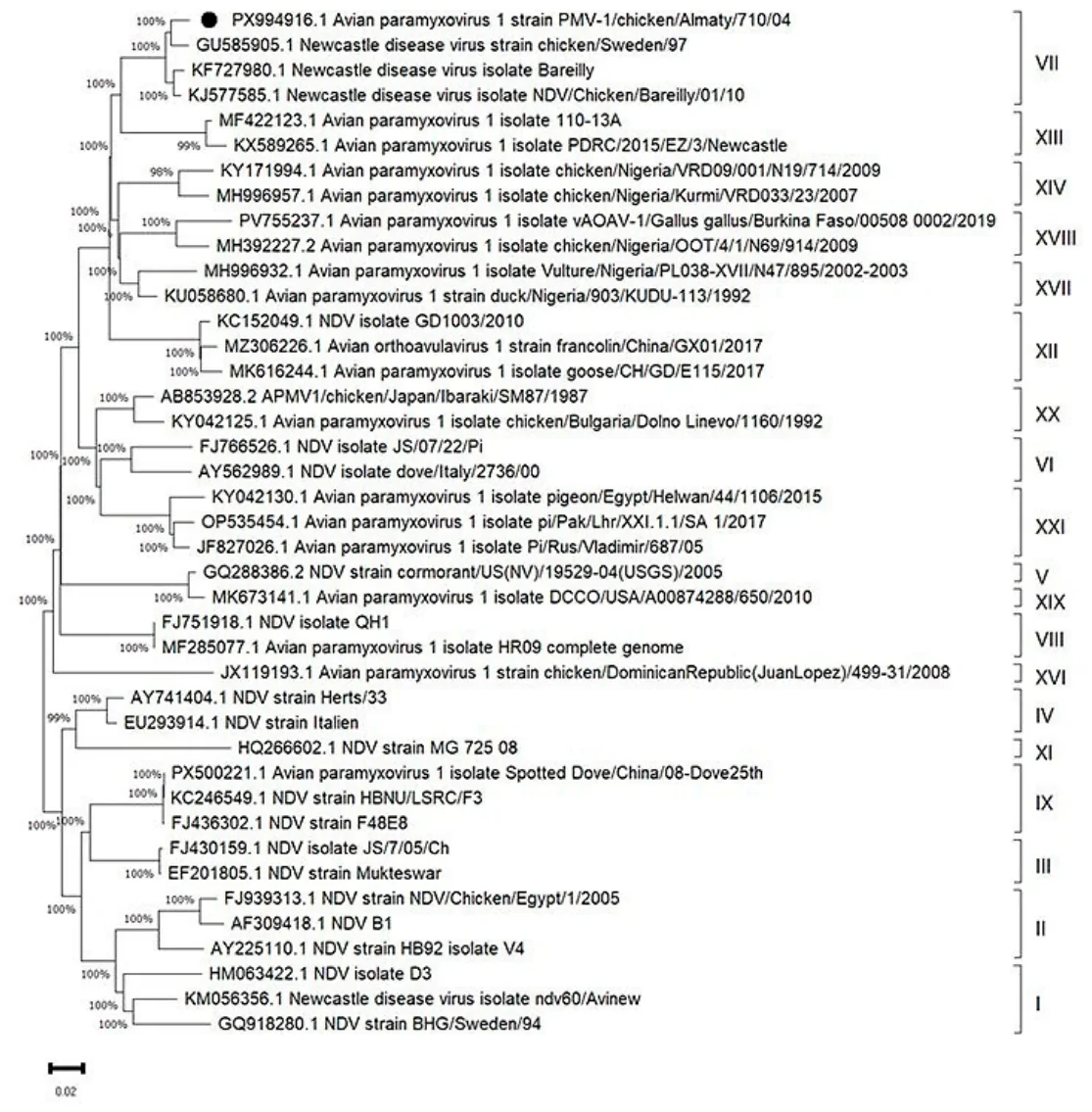
Phylogenetic analysis of the PMV-1/chicken/Almaty/710/04 strain (marked with a black circle) along with 40 global strains of various representatives of the genus Orthoavulavirus obtained from the NCBI GenBank database. The *x*-axis represents the scale of the tree.

Based on phylogenetic analysis (Figure 3), the investigated strain ‘PMV-1/chicken/Almaty/710/04’ was found to belong to class II, genotype VII, harboring the RRQKRF amino acid motif in the F gene, which indicates its high virulence. It formed a monophyletic clade with a bootstrap value of 100% together with the sequences GU585905.1 (strain chicken/Sweden/97), KF727980.1 (Bareilly isolate), and KJ527585 (isolate NDV/Chicken/Bareilly/01/10). BLASTN analysis (NCBI) revealed that the investigated isolate exhibited a high degree of nucleotide sequence identity (97.76%) with the Swedish isolate (GU585905.1) and the Indian isolates (97.4% and 97.3%, respectively).

### 3.2. Physicochemical Parameters of the Vaccine

According to the analysis results, the physicochemical parameters of the emulsified vaccine fully complied with the established specifications. The pH of the inactivated vaccine was 7.23 ± 0.00, which meets the requirements for neutrality and physiological compatibility. Microbiological testing via plating on selective and enriched culture media confirmed the sterility of the preparation: no growth of bacterial or fungal flora, nor mycoplasmas, was detected.

Visual inspection after thermostating revealed no signs of emulsion phase separation (aqueous phase separation), indicating the high stability of the oil phase.

Storage stability studies demonstrated that the preparation retains its properties without significant changes for 3 months at 25 °C and up to 12 months at 4 °C. The kinematic viscosity values were 38.62 ± 0.01 and 39.20 ± 0.01 mm^2^/s, respectively, which fall within the acceptable range of 20–150 mm^2^/s.

Collectively, the obtained data confirm the stability of the dosage form, the quality of the inactivation process, and the vaccine’s compliance with safety criteria for further evaluation of its immunogenic activity.

### 3.3. Safety Evaluation of the Vaccine Formulation

During the 10-day post-vaccination observation period, no mortality or clinical abnormalities were recorded in the experimental chicks. Post-mortem examination revealed no macroscopic changes (edema, hyperemia, necrosis, or residual vaccine) in the muscle tissue at the injection sites. These findings demonstrate the high local and systemic tolerability of the vaccine.

### 3.4. Evaluation of the Vaccine’s Ability to Induce a Specific Immune Response

**Figure 4:**
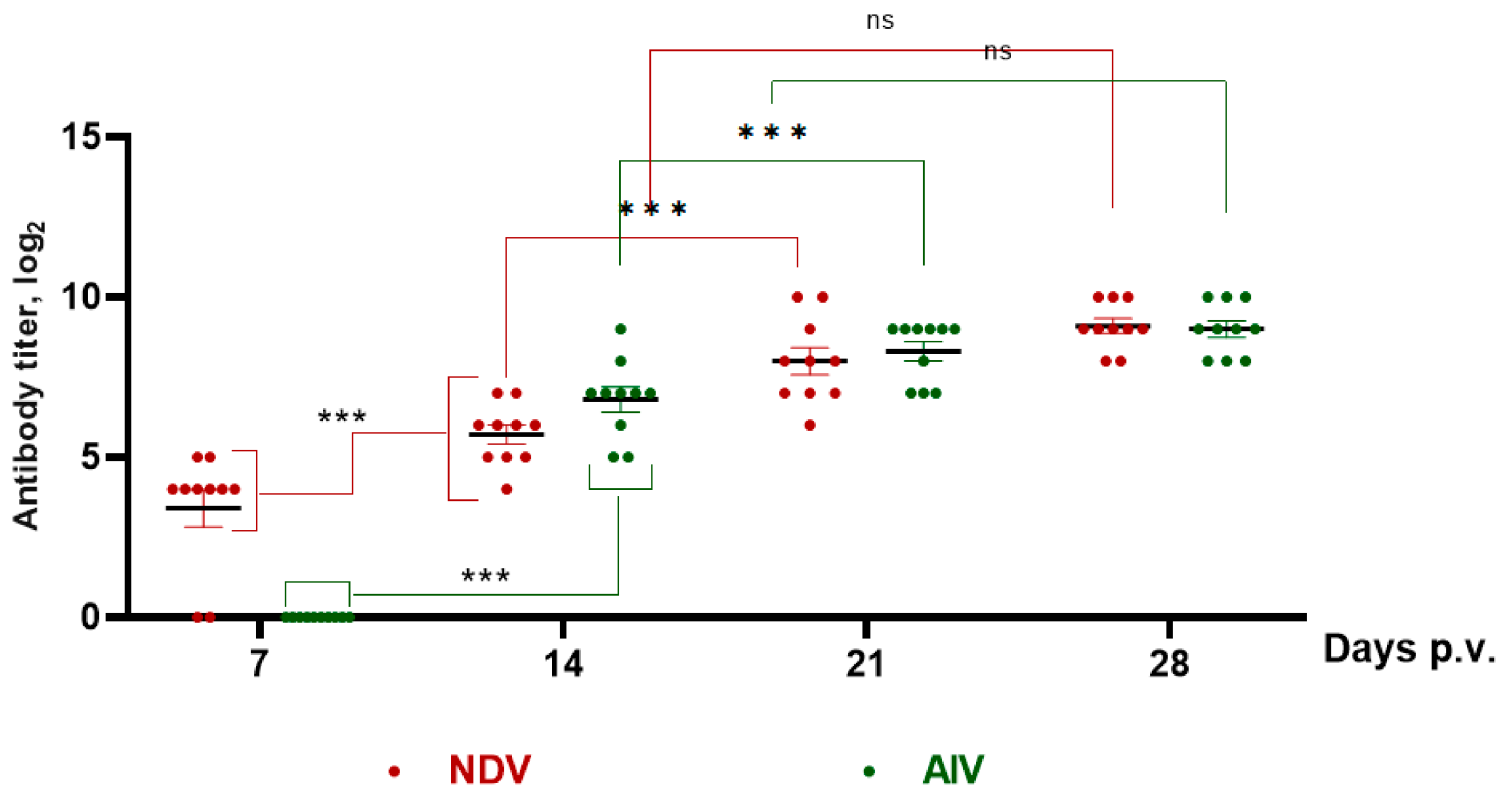
Dynamics of antibody titers against Newcastle disease virus (NDV) and avian influenza virus (AIV) in immunized chickens. Serum samples were collected at 7, 14, 21, and 28 days post-vaccination (p.v.). Antibody titers were determined by the hemagglutination inhibition (HI) test and expressed as log_2_ values. Data are means ± standard errors; *** indicates *p* < 0.0005, and ns indicates no significant difference (*p* > 0.05).

One of the primary objectives of this study was to evaluate the capacity of the developed vaccine to induce a specific immune response against Newcastle disease virus and avian influenza virus. The immunogenicity of the vaccine was evaluated in experimental chicks, followed by the determination of specific antibody levels and the assessment of protective efficacy. The kinetics of the immune response are presented in Figure 4, and the individual antibody titer data for all birds are provided in Supplementary Table S1.

Analysis of the data presented in Figure 4 showed that during the early post-vaccination period, chicks in the experimental group exhibited a moderate level of antibodies against the Newcastle disease virus, with a mean log_2_ titer of 3.40 ± 0.58, whereas antibodies against the avian influenza virus were undetectable. The differences between the antibody titers to the two antigens were highly significant (*p* < 0.0001), indicating an earlier onset of the immune response to the Newcastle disease component.

Subsequently, a significant increase in specific antibody titers against both viruses was observed over time. By day 14, the mean log_2_ titers reached 5.70 ± 0.30 for Newcastle disease and 6.80 ± 0.39 for avian influenza. By day 21, they increased to 8.00 ± 0.42 and 8.30 ± 0.30, respectively. Importantly, no statistically significant differences were observed between the antibody titers to the two antigens at days 14, 21, and 28 (*p* > 0.05), demonstrating a highly synchronized development of the humoral immune response to both vaccine components after the initial phase.

By the end of the observation period (day 28), the maximum level of specific antibodies in the experimental group was achieved for both antigens, reaching 9.10 ± 0.23 log_2_ for NDV and 9.00 ± 0.26 log_2_ for AIV (*p* > 0.05). This result confirms a balanced immune response to both antigens in the combined vaccine and supports the appropriateness of the selected antigen load.

In the control group, specific antibodies against the Newcastle disease virus and the avian influenza virus were not detected throughout the entire study period, which rules out background virus circulation and confirms the specificity of the recorded immune response.

### 3.5. Evaluation of Vaccine Protective Efficacy

**Figure 5:**
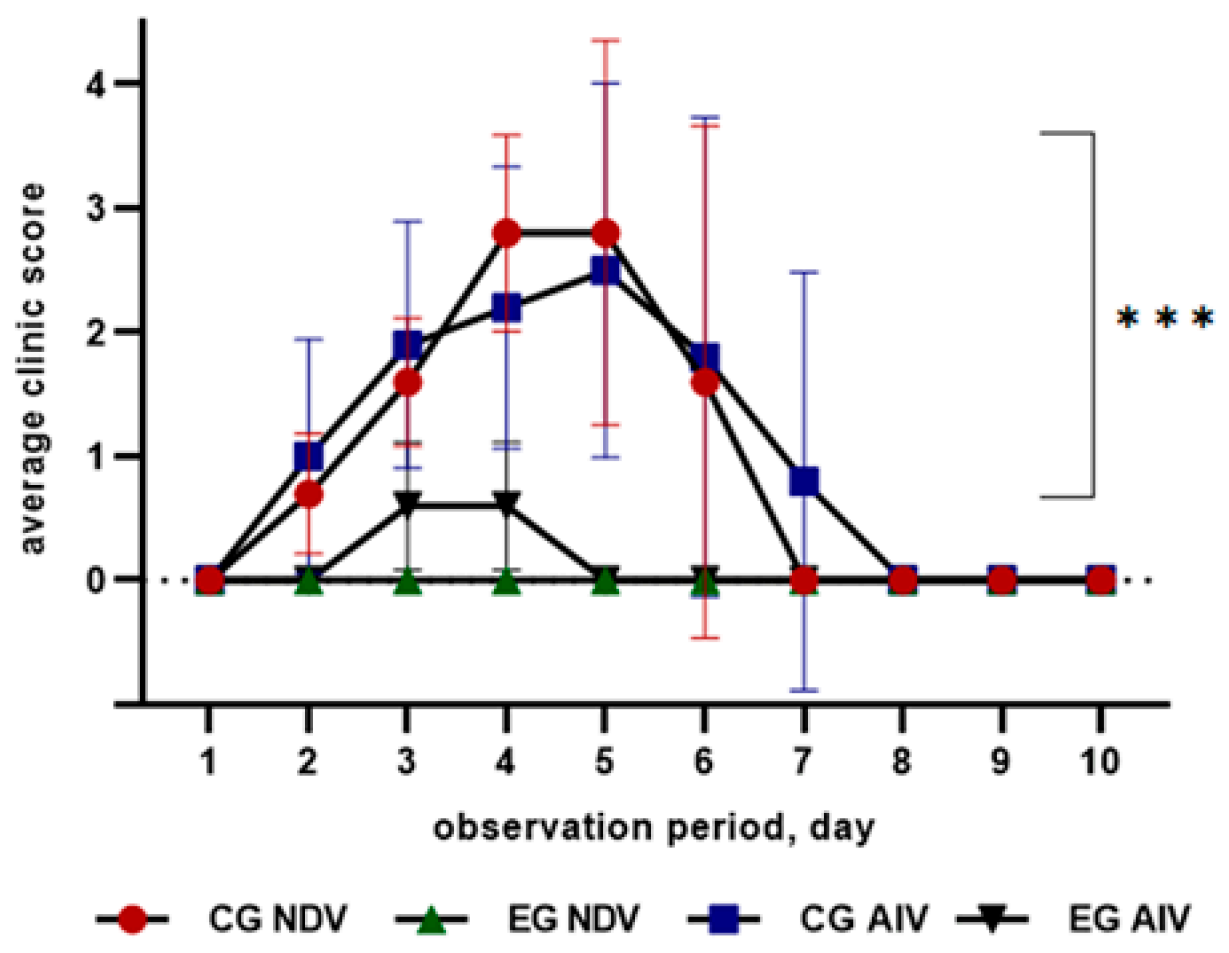
Clinical sign severity in vaccinated and control chickens following challenge with virulent strains. Note: EG NDV—vaccinated group, challenged with NDV; CG NDV—control group, challenged with NDV; EG AIV—vaccinated group, challenged with AIV; CG AIV—control group, challenged with AIV; NDV—Newcastle disease virus; AIV—avian influenza virus. Data are means ± standard errors; indicates *** (*p* < 0.0001, and ns indicates no significant difference (*p* > 0.05).

The protective efficacy of the vaccine was evaluated by challenging chicks with virulent epizootic isolates of NDV and AIV. The survival dynamics and the severity of clinical signs in the experimental and control groups are presented in Figure 5.

In the control groups challenged with Newcastle disease virus and avian influenza virus, 100% mortality was observed, confirming the high virulence of the challenge strains. Following NDV challenge, mortality began on days 4–5 post-inoculation and reached 100% by day 6, accompanied by a sharp increase in the mean clinical score to 4. In the group challenged with the avian influenza virus, fatalities were recorded starting from days 3–4, with 100% mortality occurring by day 7 of observation.

In the experimental (vaccinated) group challenged with NDV, the clinical score remained at 0 in all birds throughout the 10-day observation period: no clinical signs of infection were observed, and no mortality was recorded, indicating absolute protective efficacy against NDV.

Following challenge with the avian influenza virus, the majority of vaccinated chicks also showed no clinical signs (score of 0). However, on days 3–4 post-inoculation, a minor proportion of the birds exhibited transient moderate depression and reduced water consumption, which was scored as 1 point. Starting from day 5, the condition of the birds completely normalized, clinical scores returned to zero, and 100% survival was maintained until the end of the observation period without any residual effects.

Analysis of viral shedding revealed that in the control groups, the presence of the Newcastle disease and avian influenza viruses was detected at high titers (6.5–8.0 log_10_ EID_50_/mL) in tracheal and cloacal swabs throughout the entire observation period until the death of the birds. In the experimental (vaccinated) group, Newcastle disease virus was undetectable as early as day 4 post-challenge, whereas avian influenza virus was detected in a few birds up to day 3 at low titers (≤2.0 log_10_ EID_50_/mL), after which it was completely eliminated. The obtained data demonstrate the ability of the developed vaccine not only to prevent clinical manifestations of the infection but also to significantly reduce viral shedding, which is critical for breaking epizootic chains and limiting the horizontal spread of pathogens in poultry farms.

## 4. Discussion

In the present study, a combined inactivated vaccine against highly pathogenic avian influenza (subtype H5N8, clade 2.3.4.4b) and Newcastle disease (genotype VII) was developed and comprehensively evaluated based on current epizootic strains circulating in Kazakhstan. Our phylogenetic analysis confirmed that the NDV strain “PMV-1/chicken/Almaty/710/04” used in this study belongs to the highly virulent genotype VII, which currently dominates in Eurasia and the Commonwealth of Independent States (CIS) [7], providing the rationale for utilizing this field isolate as an antigenic component. Genotype VII NDVs circulating in the region over the past two decades demonstrate a high degree of genetic conservation in the key antigenic sites of the F and HN glycoproteins. Comparative analysis reveals that the amino acid sequence of the F protein of this strain shares over 96% similarity with recent genotype VII isolates from Kazakhstan and other CIS countries [27]. Since protective immunity against Newcastle disease is primarily mediated by antibodies against the F and HN proteins, the use of this endemic strain ensures reliable cross-protection against currently circulating variants. This approach addresses the genotype mismatch issue associated with traditional live vaccines based on classical strains (such as LaSota or Hitchner B1) and provides homologous antigenic protection [30,31]. The necessity of including the homologous highly pathogenic avian influenza virus strain “A/Wild goose/Kostanay/KZ/83/2021” (subtype H5N8) in the combined vaccine is driven by the global expansion of clade 2.3.4.4b (including subtypes H5N8 and H5N1) [32]. This strategy aims to ensure reliable protection against the persistent threat of virus reintroduction from wild and synanthropic bird populations into commercial farms, which is particularly relevant for endemic regions with active migration routes [3,6]. Thus, the developed combined vaccine directly addresses the challenges of the current epizootic situation, offering a scientifically grounded solution for comprehensive prophylaxis under conditions of simultaneous circulation of both pathogens.

A key methodological challenge in the development of combined inactivated vaccines is potential antigenic interference—the phenomenon of suppression of the immune response to one of the components during co-immunization. Nevertheless, in our experiments, a synchronous and balanced increase in antibody titers against both antigens was achieved, reaching 9.10 ± 0.23 log_2_ for NDV and 9.00 ± 0.26 log_2_ for AIV by day 28, indicating the absence of negative interactions. Our results are consistent with recent international studies. For example, [8] reported no significant antigenic interference when evaluating bivalent inactivated vaccines against H5N8 and NDV, attributing this to the use of high-quality water-in-oil emulsions, which allow for the independent processing and presentation of both antigens. Furthermore, [9] emphasized that combined vaccines induce a more balanced humoral immune response compared to the separate administration of monovalent vaccines. The choice of the Montanide™ ISA 78 VG adjuvant system, which forms a water-in-oil (W/O) emulsion with a 30:70 ratio, played a crucial role in this outcome. According to the classification and mechanisms of action described by [33], water-in-oil emulsions ensure the formation of an antigen depot at the injection site, prolonged antigen release, and potent activation of both the humoral and cellular arms of the immune system. A recent review [34] confirms that properly selected new-generation oil adjuvants are capable of overcoming immune tolerance and inducing high titers of neutralizing antibodies even when multiple antigens are administered simultaneously.

The favorable tolerability of the chosen adjuvant system is further supported by the absence of local reactions, maintenance of body weight, and normal behavior in the vaccinated birds. This aligns with the conclusions of [35] that modern water-in-oil adjuvants based on highly purified mineral oils are characterized by a favorable safety profile and comparable immunostimulatory activity to more reactogenic systems.

The challenge results unequivocally confirmed the high protective activity of the vaccine. The 100% mortality in the unvaccinated control groups (death occurring on days 4–7) demonstrates the pronounced virulence of the challenge strains and the complete susceptibility of the birds. At the same time, complete clinical protection and the absence of symptoms in the immunized group meet international criteria. The data obtained in our study regarding the significant reduction in viral shedding in vaccinated birds, along with the achievement of high antibody titers (≥8.0 log_2_), confirm the ability of the developed vaccine not only to provide clinical protection but also to limit the horizontal spread of pathogens. Our data on the reduction in the viral load to a subclinical level are consistent with the findings of [12,36], who demonstrated that homologous inactivated oil-based vaccines significantly reduce viral shedding titers, which is critical for breaking epizootic chains [37].

Collectively, the obtained data confirm the validity of the design rationale for this bivalent vaccine as a promising candidate for commercial application, offering a reliable and scalable strategy to overcome the limitations of traditional monovalent vaccination in endemic regions.

This study has several limitations that should be considered when interpreting the results. First, although the size of the experimental group is sufficient to demonstrate basic protective efficacy under laboratory conditions, it is insufficient to detect rare adverse reactions or broader population-level effects. Second, the 10-day observation period post-challenge—while standard for assessing acute mortality in HPAI and ND—does not allow for a comprehensive evaluation of the long-term consequences of subclinical infections. Third, this study did not evaluate the duration of post-vaccination immunity or the effect of vaccination on the reproductive performance of poultry. Further research, including the evaluation of the duration of immunity (for at least 6 months), is necessary to confirm these results and substantiate their future commercial application.

## 5. Conclusions

Based on the whole-genome characterization of an epizootic Newcastle disease virus strain (genotype VII) and considering the current circulation of the avian influenza virus (H5N8, clade 2.3.4.4b), a production technology for a combined inactivated vaccine formulated with the Montanide™ ISA 78 VG adjuvant has been developed.

It has been demonstrated that the vaccine induces a robust, synchronous, and balanced humoral immune response. The absence of antigenic interference is confirmed by the achievement of peak specific antibody titers (9.10 ± 0.23 log_2_ for NDV and 9.00 ± 0.26 log_2_ for AIV) against both antigens by day 28 post-immunization.

The high protective efficacy of the combined vaccine has been experimentally proven, providing 100% clinical protection and survival in chicks following challenge with virulent epizootic isolates of the Newcastle disease virus and avian influenza virus.

The practical significance of this study lies in the fact that the developed combined vaccine represents a promising and cost-effective candidate for comprehensive specific prophylaxis. Its potential implementation could optimize immunization regimens, reduce the frequency of bird handling, and mitigate economic losses in regions facing complex epizootic scenarios involving both avian influenza and Newcastle disease.

## Data Availability

The raw data supporting the conclusions of this article, including individual animal antibody titers, will be made available by the authors as Supplementary Table S1, without undue reservation.

## Supplementary Materials

The following supporting information can be downloaded at: https://www.mdpi.com/article/10.3390/vaccines14080669/s1, Table S1: Individual and mean HI antibody titers (log_2_) in birds after vaccination.
