# Supplementary material for: Development, Immunogenicity and Protective Efficacy of an Associated Inactivated Vaccine Against Highly Pathogenic Avian Influenza and Newcastle Disease in Chickens
Source: Vaccines (Basel). 2026 Aug 1;14(8):669. doi: 10.3390/vaccines14080669 (PMC13517504; doi:10.3390/vaccines14080669)
Supplement: Supplementary file 1 [file vaccines-14-00669-s001.zip › vaccines-4439503-supplementary.pdf]

Supplementary Table S1: Individual and mean HI antibody titers (log_2) in birds after vaccination

Bird No.	Day 7 (Titer)	Day 7 (log_2)	Day 14 (Titer)	Day 14 (log_2)	Day 21 (Titer)	Day 21 (log_2)	Day 28 (Titer)	Day 28 (log_2)
**Newcastle Disease Virus (NDV) Component**								
**1**	0	0	20	4	160	7	640	9
**2**	0	0	40	5	80	6	320	8
**3**	20	4	80	6	640	9	1280	10
**4**	20	4	80	6	160	7	640	9
**5**	40	5	40	5	320	8	640	9
**6**	20	4	160	7	160	7	320	8
**7**	40	5	80	6	320	8	640	9
**8**	20	4	80	6	1280	10	1280	10
**9**	20	4	160	7	1280	10	1280	10
**10**	20	4	40	5	320	8	640	9
**Mean ± SEM**	-	3.40 ± 0.58	-	5.70 ± 0.30	-	8.00 ± 0.42	-	9.10 ± 0.23
**Avian Influenza Virus (AIV) Component**								
**1**	0	0	160	7	320	8	640	9
**2**	0	0	160	7	160	7	320	8
**3**	0	0	160	7	640	9	640	9
**4**	0	0	80	6	640	9	1280	10
**5**	0	0	640	9	640	9	640	9
**6**	0	0	320	8	640	9	640	9
**7**	0	0	160	7	640	9	1280	10
**8**	0	0	40	5	160	7	320	8
**9**	0	0	40	5	160	7	320	8
**10**	0	0	160	7	640	9	1280	10
**Mean ± SEM**	-	0.00	-	6.80 ± 0.39	-	8.30 ± 0.30	-	9.00 ± 0.26

Note: Antibody titers were determined by Hemagglutination Inhibition (HI) assay starting at a 1:10 dilution.

- Undetectable antibody titers (< 1:10) were assigned a value of 0 for both raw titers and log_2 scale representation.
- Data are presented as individual values and Mean log_2 titer ± Standard Error of the Mean (SEM).
